# Intestinal Parasitic Infections in HIV Infected and Non-Infected Patients in a Low HIV Prevalence Region, West-Cameroon

**DOI:** 10.1371/journal.pone.0057914

**Published:** 2013-02-25

**Authors:** Céline Nguefeu Nkenfou, Christelle Tafou Nana, Vincent Khan Payne

**Affiliations:** 1 Laboratory of Systems Biology, “Chantal Biya” International Reference Centre for Research on HIV/AIDS Prevention and Management (CBIRC), Yaounde, Cameroon; 2 Department of Biological Sciences, Higher Teacher Training College, University of Yaounde I, Yaounde, Cameroon; 3 Department of Animal Biology, Faculty of Science, University of Dschang, Dschang, Cameroon; Fundacion Huesped, Argentina

## Abstract

The magnitude of intestinal parasitic infection in acquired immunodeficiency syndrome patients requires careful consideration in the developing world where poor nutrition is associated with poor hygiene and several tropical diseases. However, there have been very few studies addressing this issue in Cameroon. This study was conducted to determine the prevalence of intestinal parasitosis in HIV/AIDS patients in Dschang -Cameroon. Stool and blood specimens from HIV/AIDS patients and control group were screened respectively for intestinal parasites and for HIV antibodies. Intestinal parasites were identified using direct microscopy, formalin-ether concentration and Ziehl Neelsen methods. Out of 396 participants recruited among patients consulting at hospital, 42 (10.6%) were HIV positive, thirty of them treatment naïve. The overall prevalence of intestinal parasites was 14.64%. Out of 42 HIV/AIDS patients, 59.5% (25/42) were infected with intestinal parasites, while only 9.32% (33/354) of the HIV negative patients were infected with intestinal parasites. The parasites detected in our study population included *Crystosporidium parvum* (2.53%), *Entamoeba histolytica* (7.52%), *Entamoeba coli* (4.04%), *Giardia lamblia* (0.25%), *Trichuris trichura* (0.25%), *Strongyloides stercoralis* (0.25%) and *Taenia spp*. (0.25%). In the HIV infected group, *Crystosporidium parvum* (19.04%), *Entamoeba histolytica* (19.04%), *Entamoeba coli* (21.42%), *Giardia lamblia* (2.38%), *Strongyloides stercoralis* (0.25%) and *Taenia* spp. (0.25%) were found. *Crystosporidium parvum* was found to be significantly higher in HIV/AIDS patients than in controls (P<0.05). Multivariate analysis showed that the HIV status and the quality of water were the major risk factors for intestinal parasitosis. Routine examinations of stool samples for parasites would significantly benefit the HIV patients by contributing in reducing morbidity and improving the efficiency of antiretroviral treatment. Even after the introduction of free anti-retroviral drugs, opportunistic intestinal infections are still a threat. HIV patients should be screened routinely for intestinal parasites and treated for their overall well being.

## Introduction

Intestinal parasites are more common in places where poor sanitation reigns, especially in developing countries of the tropics. They can be more aggressive in children and the elderly than in middle-aged people and in immunocompromised patients.

One of the main features of Human Immunodeficiency Virus (HIV) infection is immunosuppression that leads to exposing the subject to a variety of microbial and parasitic attacks. Moreover in the tropics, there is a consistent association between HIV infection and other diseases including malaria, Mycobacterium tuberculosis and intestinal parasitosis [1]. These infections (opportunistic or not) were prevalent worldwide [2]. The infection rate is remarkably high in Sub-Saharan Africa where most cases are common among HIV positive patients. The incidence of parasitic infections was 50% in developed countries while it reached 95% in developing countries [3]. Immunosuppression, a consequence of HIV infection favors the occurrence of multiple opportunistic infections responsible for a high mortality [4], the foremost gastroenteritis occupy a significant place [5]. Among these diseases, intestinal parasites are the main cause of severe chronic diarrhea [4]. Coccidia (*Cryptosporidium parvum*, *Isospora belli*, *Cyclospora sp*) and amoebae (*Entamoeba histolytica*, etc.) are the etiologic agents commonly responsible for the genesis of these intestinal protozoans in HIV-positive persons in many parts of the world [6]. During the evolution of HIV infection, the gastrointestinal involvement is frequent and 90% of patients consult for gastrointestinal disorders [7]. Associations of HIV and opportunistic intestinal parasites can be of three kinds: – a simple consequence of immunodeficiency, – be caused by a specific interaction at the intracellular level, when co-infection of the same type of cell or intercellular through various mediators – finally, an association to a cyclical confounding iatrogenic [1]. The clinical spectrum caused by these parasitic protozoa particularly among HIV positive patients ranges from asymptomatic infection to severe infection (chronic diarrhea, dehydration and mal-absorption) [8].

Such co-infections present with more severe clinical symptoms compared to parasite infections of otherwise healthy people, and are more difficult to treat [9].

These parasites have an undeniable influence on the patient's general condition. Their frequency varies with the risk factors and the geographical origin of patients [10]. To fight against opportunistic infections there are well codified treatment regimes with good prognosis [11], [12]. From these facts, we strongly believe that the study of the prevalence and etiologic spectrum of parasitic infections in patients with HIV is necessary to understand the severity or progression of the disease HIV/AIDS (Acquired Immuno Deficiency Syndrome) its consequences in the effectiveness of anti-retrovirals (ARVs) and to make the best decision for the care of people living with HIV/AIDS (PLWHA). Parasite – HIV co-infections are one of the neglected areas in HIV research in Cameroon although HIV generally has become a major public health concern in Cameroon and beyond. Even if the concerns regarding opportunistic parasitic infections among HIV positives have been widely recognized, only few relevant field-epidemiological investigations have been reported in Cameroon. Two similar studies, one in Yaounde and the other in Douala [13], [14] respectively have been done. In the district of Dschang, West Region of Cameroon, very little data exist on the prevalence and impact of intestinal infections in HIV-positive patients.

We carried out a parasitological survey among HIV infected and HIV non infected patients consulting in two hospitals in Dschang, West Region, Cameroon to understand the epidemiological situation and risk factors of co-infection of HIV and intestinal parasites, five years after the introduction of free ARV in Cameroon and in a low HIV prevalence setting. The ultimate goal of the study was to provide guidance on the prevention and control of co-infections including treatment needs of HIV/AIDS patients [15], and thus decrease the adverse effects of intestinal parasites on people living with HIV.

## Methods

### Ethical statement

The study protocol was approved by the “Cameroon National ethics committee” (CNE) under the registration N° 269/CNE/SE/2011.

Participants consulting at the hospitals were kindly requested by the study team to participate in the study. Interested individuals provided written informed consent. All participants were offered professional counseling before and after HIV testing for those who had never done their test before. All diagnostic results were kept strictly confidential. Deworming treatments (albendazole, praziquantel) were proposed to all participants found to be infected with helminths.

### Study area and population

The study was conducted in the district hospital and the Saint Vincent de Paul hospital in Dschang city. Dschang is a small city in the west Region of Cameroon with the regional prevalence of HIV decreasing from 4.7% in 2004 to 2.8% in 2011. The West Region is among the Regions of low HIV prevalence in Cameroon.

### Process of the survey

Patients were enrolled from March 2012 through July 2012. Patients presenting at the hospitals were requested to participate in the study. After their consent, a questionnaire was administered to each participant by the nurse who had been specifically trained for this task. A pair sample of stool and blood were collected from each of the participants and used for intestinal parasites and HIV testing respectively. Since there is a likelihood of HIV misdiagnosis, HIV testing was performed for all our study participants to confirm their positivity.

### Laboratory procedures

The blood samples of all participants were screened for anti-HIV antibodies using Determine HIV 1/2 HIV rapid test (Alere, Chiba, 270–2214, Japan). Reactive samples were subjected to confirmation using Genie III 1/2 HIV rapid test (Bio-Rad, 92430 Marnes-la-coquette, France). Samples reactive to both HIV-1 and HIV-2 were tested for the presence of HIV-2 using specific primer in a polymerase chain reaction (PCR). Rapid tests were conducted in the district hospital and confirmation of HIV-2 was done at the “Chantal Biya” International Reference Centre for Research on HIV prevention and management (CBIRC) in Yaounde.

Stool samples were analyzed by direct microscopy with physiological saline and iodine, and after concentration by the formalin ether method coupled with the Ziehl Neelsen modified technique for the detection of *Cryptosporidium sp* and *Isospora belli* [16]. The identification of *Cryptosporidium* and *Isospora* was done using Ziehl Neelsen method which somehow presents a disadvantage over the Kinyoun technique also called cold Ziehl Neelsen technique [17], [18]. In fact the Ziehl Neelsen technique requires the heating of fuchsin that produces toxic and carcinogenic vapors. Based on the availability of reagents, and the performance for parasites staining, we used the Ziehl Neelsen technique.

### Statistical analysis

Data were registered in Microsoft excel 2010 and analyzed with Statistical Package for Social for the Science (SPSS) version 11.0 statistical software. Chi square (x^2^) test allowed us to compare the prevalence of intestinal parasites according to age and sex. Multivariate logistic model was used to evaluate the risk of parasitic infection according to HIV status, socio demographic characteristics and hygiene condition. We have also tested the parasitic infection density according to the HIV sero-status using Mann-whitney test. Associations were tested at 95% confidence.

## Results

### Study cohort

A total of 396 people were recruited. 148 (37.37%) were male and 248 (62.62%) were female. Complete data were collected from all participants who had provided stool and blood samples as well as answered the questionnaires. The summary of these data are presented in table 1. Sex ratio M/F was 0.6 (2/3: about 2 male for 3 female).

**Table 1:** Overall population demographic and clinical data.

Variable	Overall Population	HIV/AIDS patients	infected with protozoans/Helminths
**Sex**			
Male	148 (37.37%)	8 (19.04%)	15 (25.86%)
Female	248 (62.62%)	34 (80.95%)	43 (74.13%)
**Average age (year)**	29.3	33.26	31.08
**Marital status**			
Single	200 (50.50%)	13 (30.85%)	22 (37.93%)
Married	181 (40.70%)	28 (66.66%)	32 (55.17%)
Concubin	14 (3.53%)	1 (2.38%)	4 (6.89%)
Widowed	1 (0.25%)	0	0
**Education level**			
Illiterate	4 (1.01%)	1 (2.38%)	1 (1.72%)
Primary school	65 (16.41%)	12 (28.57%)	17 (29.31%)
Secondary school	138 (34.84%)	21(50%)	22 (78.57%)
Post Secondary school	199 (50.25%)	8 (19.04%)	18 (31.03%)
**Occupation**			
Student	187 (47.22%)	8 (19.04%)	20 (34.48%)
Farming	8 (2.02%)	1 (2.38%)	1 (1.72%)
Others	144 (36.36%)	17 (40.47%)	23 (39.65%)
Unemployed	57 (14.39%)	16 (38.09%)	14 (24.13%)
**Personal hygiene**			
**Wash of hands**			
Always	123 (31.06%)	9 (21.42%)	19 (32.75%)
Sometimes	258 (65.15%)	30 (71.42%)	37 (63.79%)
Not at all	15 (3.76%)	3 (7.14%)	2 (3.44%)
**Nail cleaning**			
Always	180 (45.45%)	15 (35.71%)	25 (43.10%)
Sometimes	213 (53.78%)	26 (61.90%)	32 (55.17%)
Not at all	3 (0.75%)	1 (2.38%)	1 (1.72%)
**Cleaning after defecation**			
Wash with water	48 (12.12%)	5 (11.90%)	4 (6.89%)
Use toilet paper	282 (71.21%)	28 (66.66%)	43 (74.13%)
Others methods	66 (16.66%)	9 (21.42%)	11 (18.96%)
**Source of water consumed**			
Well	4 (1.01%)	2 (4.76%)	1 (1.72%)
Borehole (forage)	42 (10.60%)	2 (4.76%)	1 (1.72%)
Spring (source)	128 (32.32%)	10 (23.80%)	18 (31.03%)
Tap	133 (33.58%)	15 (35.71%)	18 (31.03%)
Bottled water	8 (2.02%)	1 (2.38%)	0
> or = two propositions	81 (20.45%)	12 (28.57%)	20 (34.48%)

Most of the participants were consulting for abdominal pain and fever 17. 67%.

Patients recruited in the study were aged 15 to 65 with the mean being 30 years.

### HIV testing

Forty-two (42) patients were HIV positive and 354 HIV negative, giving a prevalence of 10.6%: of these, eight (19.05%) were male and 34 (80.95%) were female. Furthermore, three HIV-2 cases were confirmed using primer specific PCR. Twelve HIV infected patients were on ARV treatment, while 30 were treatment naive.

### Parasitic infections

The overall prevalence of intestinal protozoa infections was 14.64% (58/396).

Considering the HIV serological status, we had among the HIV negative patients 9.32% (33/354) and among HIV positive 59.52% (25/42) parasite prevalence. As summarized in table 2, HIV infected patients were more likely to be infected with intestinal parasites than HIV non-infected patients (P<0.0001). With respect to sex (see table 2), we had 15/148 males infected with intestinal parasites while 43/248 females were infected. Females were more likely to be infected than males with a P value of 0.049.

**Table 2:** Prevalence of intestinal parasites according to age and sex.

	Overall population		HIV/AIDS patients		Infected with protozoans/Helminths	
Age range (years)	Male	Female	Male	Female	Male	Female
15–30	98 (66.21%)	155 (62.5%)	3 (37.5%)	12 (35.29%)	5 (33.33%)	25 (58.13%)
>30	50 (33.78%)	93 (37.5%)	5 (62.5%)	22 (64.70%)	10 (66.66%)	18 (41.86%)
**Total**	**148**	**248**	**8**	**34**	**15**	**43**

According to age (see table 2), grouped as 15–30 and more than 30 years old, older participants were more likely to be infected by intestinal parasites. P value of 0.036 was obtained.

The parasites detected in our study population included *Crystosporidium parvum* (2.53%), *Entamoeba histolytica* (7.52%), *Entamoeba coli* (4.04%), *Giardia lamblia* (0.25%), *Trichuris trichura* (0.25%), *Strongyloides stercoralis* (0.25%) and *Taenia spp*. (0.25%).

Globally, most frequently observed parasite in the entire study population was *Entamoeba histolytica* (7.52%), while specifically in HIV infected patients, *Cryptosporiduim parvum* (19.04%) and *Entamoeba histolytica* (19.04%) were the most recorded pathogenic parasites as seen in table 3. *Isospora belli*, a commonly reported parasite in such studies was not identified in our study population.

**Table 3:** Summary of the clinical data of the HIV infected patients.

Patients (n)	Sex	Age (ans)	EDEF^i^	CZNM^ii^	Virus	DARViii
1	F	30	*E. coli*	*/*	HIV-1	1 month
1	M	23	*/*	*C.* *parvum*	HIV-1/2	Naive
1	F	21	*/*	*C.* *parvum*	HIV-1/2	Naive
1	F	32	*/*	*C.* *parvum*	HIV-1	1 month
1	F	35	*E.* *hystolitica*	*/*	HIV-1	Naive
1	M	42	*E.* *hystolitica*	*/*	HIV-1	Naive
1	M	37	*E.* *hystolitica*	*/*	HIV-1	Naive
1	F	24	*E.* *coli*	*/*	HIV-1	Naive
1	F	26	*/*	*C.* *parvum*	HIV-1	7 days
1	F	22	*E.* *hystolitica*	*/*	HIV-1	Naive
1	M	50	*E.* *hystolitica*	*/*	HIV-1	Naive
1	F	53	*/*	*C.* *parvum*	HIV-1	5 years
1	F	40	*/*	*C.* *parvum*	HIV-1	1 year
1	F	35	*E. coli*	*/*	HIV-1	1 day
1	F	37	*E.* *hystolitica*	*/*	HIV-1	Naive
1	F	21	*E. coli*	*/*	HIV-1	Naive
1	F	25	*E. coli*	*/*	HIV-1	6 days
1	F	28	*/*	*C.* *parvum*	HIV-1	3 years
1	F	32	*E. coli*	*C.* *parvum*	HIV-1	3 years
1	F	34	*E. coli*	*/*	HIV-1	1 year
1	F	31	*E. coli*	*/*	HIV-1	Naive
1	F	38	*G.* *lamblia*	*/*	HIV-1	7 days
1	F	35	*E.* *hystolitica*	*/*	HIV-1	Naive
1	M	38	*E. coli*	*/*	HIV-1	Naif
1	F	63	*E.* *hystolitica*	*/*	HIV-1/2	14 days
3	M	18,24,40	/	*/*	HIV-1	Naive
3	F	26,31,39	/	*/*	HIV-1	Naive
11	F	21–52	/	*/*	HIV-1	Naive

i EDEF Direct Exam with Physiological water and lugol.

ii CZNM Coloration with modified Ziehl-Neelsen.

iii Duration of the ARV treatment.

### Multi-parasitism

Multi-parasitism was rare in our population. In the HIV infected group, most participants were infected by only one parasite (23 out of 42 or 54.76% of the parasite infected HIV positive individuals), while 2 (4.76%) were infected with two species.

### Univariate and multivariate analyses

In univariate analysis, the sex, the matrimonial status, the level of education, the source of water consumed and HIV sero-status were associated with presence of intestinal parasites. Whereas, in multivariate logistics analysis, only the HIV sero-status OR = 14.0305 (7.014–29.17) and the source of water consumed were associated with the presence of intestinal parasites. The high risk was on those consuming water from mixed sources versus those consuming water from a controlled source OR = 2.41(1.2–5.15).

No statistical difference was found between HIV positive and HIV negative patients for parasitic density (P = 0.2).

## Discussion

Our study aimed at finding the epidemiology of HIV-intestinal parasites co-infection in Dschang and to evaluate the risk factors associated to this co-infection. The overall prevalence of intestinal parasites was 14.64% and was as high as 59.5% in the HIV infected patients. Our data showed that the major risk factors for intestinal parasitosis were the HIV status and the quality of water consumed.

Intestinal parasites are a diverse group of microorganisms that include single-celled protozoans and multi-cellular intestinal worms, capable of disrupting the absorption of nutrients.

*Cryptosporidium* is not transmitted when in contact with another individual, rather it is acquired through ingestion of contaminated soil or water, so respect of hygiene should be re-inforced especially in HIV patients.

Cryptosporidiosis can cause severe chronic diarrhea leading to electrolyte imbalance, mal-absorption, and profound weight loss. The physiopathology of the infection is not well understood. It is important to point out that, some studies carried out on rabbit baby model showed a reduction of ionic exchange without the intervention of prostaglandins, of trans- and paracellular transports as well as the transport of the leucine and glutamate through the ileum mucous membrane. All this resulted in the malabsorption of nutrients [19]. Non specific mechanisms such as the atrophy of villi and inflammatory reaction could also be contributing factors to this mal-absorption process. Therefore, orally taken ARV treatment will not be an exception and thus will also suffer poor absorption leading to lower ARV efficacy.

Before ARV was freely distributed to HIV patients, intestinal parasites, opportunistic or not were common in HIV patients [13]. Even after free ARV availability, intestinal parasites are still a big concern [14].

The prevalence of intestinal parasites in Cameroon varies from 33% in 2006 [13] to 27.8% in 2012 [14] and to 14.6% in the present study. Looking at the HIV infected patients group, this prevalence varies from 32% among patients with CD4 count less than 50 cells/mm^3^ in the study of Safarti *et al* in 2006 in Yaounde to 59.5% (regardless of the value of CD4 counts) in our study. This higher rate may reflect the probable severe immunodeficiency in the study population. It may also be explained by the location of our study city (Dschang) which is a small city in contrast to Yaounde, the city Capital that may have improved opportunities for good quality of water. The quality of drinking water has been the concern in the past and is still one today. Although remarkable progress has been made in the last decade by governments in providing good quality water to population, more efforts are needed in smaller cities.

The high prevalence of parasitic diseases in HIV infected patients also draws attention to the need to include routine stool examination in HIV/AIDS management.

Among the known opportunistic intestinal parasites, *Crystosporidium parvum* was encountered with the frequency of 19 .04% in HIV infected patients while only 2% were encountered in HIV negative group. These results are higher than those of Lehman *et al*, 2012 [14] obtained in Douala- Cameroon where *C. spp* was 7.4%, and those reported by Safarti *et al*., 2006 in Yaounde-Cameroon (3.9%) [13]. Strikingly, 75% of the *C. parvum* cases occurred in HIV patients under ARV treatment, and 37.5% of them had diarrhea.

From these results, we suggest that Ziehl Neelsen method of stool examination be prescribed as a routine biological examination in the management of HIV patients.

*Isospora belli* was not reported in our study. This low prevalence is in line with the study done in Yaounde [13] where only 1.9% prevalence was reported and in Congo by Wumba *et al*., 2010 where 1.7% prevalence was reported. In contrast this prevalence was as high as 5% in Thailand [20] and 7% in Brazil [21]. In two studies done in China, *I belli* was not reported [22], [23]. *Isospora belli* may not be of great public health concern in Cameroon.

One of the limitations of our study was the lack of CD4 count value of the HIV patients. It has been shown in other studies [13] that CD4 count less than 200 cells/mm^3^ was associated with higher parasitic density. More importantly, HIV patients with CD4 count less than 50 cells/mm^3^ were more at risk of opportunistic parasitic infections [14].

Also the limited sample size and the specific geographical and socio economical setting of our study, may require a larger and nationwide study in order to provide stronger recommendations.

## Conclusion

In the cross-sectional study carried out in two hospitals in Dschang-Cameroon the prevalence of HIV was found to be 10.6%. The overall prevalence of intestinal parasites was 14.6% but increased to 59.5% in HIV infected patients. HIV positive individuals are more susceptible to co-infections with *Cryptosporidium parvum* (19.04%) than HIV negative people (0.56%). In ARV treated patients the occurrence of this opportunistic intestinal parasite was higher (75%). In light of this prevalence and the damage that *C. parvum* may cause to the patient and its negative impact on the absorption of ARVs, the results obtained from this study prompt intestinal parasitic infections surveillance through the implementation of the Ziehl Neelsen or at best the Kinyoun stool examination method and their subsequent treatment. Our study also recalls that governments should continue their efforts in providing their population with good quality water.
